# Rapid sperm capture: high-throughput flagellar waveform analysis

**DOI:** 10.1093/humrep/dez056

**Published:** 2019-06-07

**Authors:** M T Gallagher, G Cupples, E H Ooi, J C Kirkman-Brown, D J Smith

**Affiliations:** 1 School of Mathematics; 2 Institute for Metabolism and Systems Research, University of Birmingham, Birmingham, UK; 3 School of Engineering, Monash University Malaysia, Bandar Sunway, Malaysia; 4 Centre for Human Reproductive Science, Birmingham Women’s and Children’s National Health Service Foundation Trust, Birmingham, UK

**Keywords:** flagellar tracking, high-throughput analysis, mathematical modelling, fluid dynamics, sperm kinematics, image analysis, computer-aided sperm analysis

## Abstract

**STUDY QUESTION:**

Can flagellar analyses be scaled up to provide automated tracking of motile sperm, and does knowledge of the flagellar waveform provide new insight not provided by routine head tracking?

**SUMMARY ANSWER:**

High-throughput flagellar waveform tracking and analysis enable measurement of experimentally intractable quantities such as energy dissipation, disturbance of the surrounding medium and viscous stresses, which are not possible by tracking the sperm head alone.

**WHAT IS KNOWN ALREADY:**

The clinical gold standard for sperm motility analysis comprises a manual analysis by a trained professional, with existing automated sperm diagnostics [computer-aided sperm analysis (CASA)] relying on tracking the sperm head and extrapolating measures. It is not currently possible with either of these approaches to track the sperm flagellar waveform for large numbers of cells in order to unlock the potential wealth of information enclosed within.

**STUDY DESIGN, SIZE, DURATION:**

The software tool in this manuscript has been developed to enable high-throughput, repeatable, accurate and verifiable analysis of the sperm flagellar beat.

**PARTICIPANTS/MATERIALS, SETTING, METHODS:**

Using the software tool [Flagellar Analysis and Sperm Tracking (FAST)] described in this manuscript, we have analysed 176 experimental microscopy videos and have tracked the head and flagellum of 205 progressive cells in diluted semen (DSM), 119 progressive cells in a high-viscosity medium (HVM) and 42 stuck cells in a low-viscosity medium. Unscreened donors were recruited at Birmingham Women’s and Children’s NHS Foundation Trust after giving informed consent.

**MAIN RESULTS AND THE ROLE OF CHANCE:**

We describe fully automated tracking and analysis of flagellar movement for large cell numbers. The analysis is demonstrated on freely motile cells in low- and high-viscosity fluids and validated on published data of tethered cells undergoing pharmacological hyperactivation. Direct analysis of the flagellar beat reveals that the CASA measure ‘beat cross frequency’ does not measure beat frequency; attempting to fit a straight line between the two measures gives ${\mathrm{R}}^2$ values of 0.042 and 0.00054 for cells in DSM and HVM, respectively. A new measurement, track centroid speed, is validated as an accurate differentiator of progressive motility. Coupled with fluid mechanics codes, waveform data enable extraction of experimentally intractable quantities such as energy dissipation, disturbance of the surrounding medium and viscous stresses. We provide a powerful and accessible research tool, enabling connection of the mechanical activity of the sperm to its motility and effect on its environment.

**LARGE SCALE DATA:**

The FAST software package and all documentation can be downloaded from www.flagellarCapture.com.

**LIMITATIONS, REASONS FOR CAUTION:**

The FAST software package has only been tested for use with negative phase contrast microscopy. Other imaging modalities, with bright cells on a dark background, have not been tested but may work. FAST is not designed to analyse raw semen; it is specifically for precise analysis of flagellar kinematics, as that is the promising area for computer use. Flagellar capture will always require that cells are at a dilution where their paths do not frequently cross.

**WIDER IMPLICATIONS OF THE FINDINGS:**

Combining tracked flagella with mathematical modelling has the potential to reveal new mechanistic insight. By providing the capability as a free-to-use software package, we hope that this ability to accurately quantify the flagellar waveform in large populations of motile cells will enable an abundant array of diagnostic, toxicological and therapeutic possibilities, as well as creating new opportunities for assessing and treating male subfertility.

**STUDY FUNDING/COMPETING INTEREST(S):**

M.T.G., G.C., J.C.K-B. and D.J.S. gratefully acknowledge funding from the Engineering and Physical Sciences Research Council, Healthcare Technologies Challenge Award (Rapid Sperm Capture EP/N021096/1). J.C.K-B. is funded by a National Institute of Health Research (NIHR) and Health Education England, Senior Clinical Lectureship Grant: The role of the human sperm in healthy live birth (NIHRDH-HCS SCL-2014-05-001). This article presents independent research funded in part by the NIHR and Health Education England. The views expressed are those of the authors and not necessarily those of the NHS, the NIHR or the Department of Health. The data for experimental set (2) were funded through a Wellcome Trust-University of Birmingham Value in People Fellowship Bridging Award (E.H.O.).The authors declare no competing interests.

## Introduction

About 100 million men worldwide are suggested to be subfertile (Inhorn and Patrizio, 2015), but for many of them an accurate diagnostic cause remains elusive. The ability of sperm to successfully migrate through the female reproductive tract is the key to natural fertilization; however, current techniques for assessing sperm motility track cell head locations without analysis of the beating flagellum, and thus lack mechanistic insight. The diagnostic power of existing measurements also remains uncertain, with studies disagreeing on the significance of existing sperm motility parameters [see Wang and Swerdloff (2014) for an overview]. The heterogeneity of human sperm requires tools that can acquire and analyse large quantities of cell data in order to achieve the statistical power necessary for gaining this insight.

Sperm motility is induced by the beating of a single flagellum which, *in vivo*, propels the cell through a complex fluidic and biochemical environment. Clinically, the current gold standard for performing a sperm motility analysis, as defined by the World Health Organization (WHO), comprises manual analysis by a trained professional (WHO, 2010). While this manual approach is excellent for an accurate sperm count or morphology analysis (on dead cells), it is widely believed that improvements to the consistency and accuracy of computer-aided analyses for motile cells would be desirable (Vander Horst, 2017; Gallagher *et al*., 2018). Indeed, it would be practically impossible to perform a manual analysis of the key features of the flagellar beat; to unlock the potential wealth of information within requires high-throughput computational tools.

Such analysis has the potential to further inform our understanding of fertilization. Changes to the beat pattern are key to many stages in the journey of a sperm: a cell must successfully transition from the relatively low-viscosity environment of semen into highly viscous cervical mucus, progress through the female reproductive tract to the oocyte and attach and detach from the epithelium. Suarez and Dai (1992) showed that hyperactivated mouse sperm are more efficient at penetrating viscoelastic media, with the resulting tracks being straighter than those of fresh sperm in the same environment. Recent modelling by Simons *et al*. (2014) has suggested that the high-amplitude beat induced by hyperactivation may be important for aiding sperm release from the epithelium.

The introduction of computer-aided sperm analysis (CASA) dates back to the 1980s. It was developed in the academic community (Holt *et al*., 1985) and progressed rapidly by commercial entities [for reviews, see Holt *et al*. (2018) and the associated special issue]. Such systems are widely used for veterinary work and in domestic animal breeding, conservation and toxicology (Amann and Waberski, 2014), but have not yet made the breakthrough into routine clinical usage. The reasons for this have been discussed elsewhere (Gallagher *et al*., 2018). Standard CASA systems produce a motility assessment from the track of the head movement; however, this does not enable causative or mechanistic insight because it lacks detail on the movement of the flagellum. Knowledge of this beat could provide hitherto untapped information for the estimation of non-visible attributes, such as the contribution of different metabolic pathways and modulation in response to the physical and biochemical environments (Ooi *et al*., 2014). The capability to capture flagellar movements and associated mechanistic insights also has broader applicability in the life sciences including the role of cilia in embryonic development (Smith *et al*., 2019), swimming of multiflagellate microorganisms (Wan and Goldstein, 2018), the use of high-speed holographic microscopy to image the flagellar waveforms of malaria parasites (Wilson *et al*., 2013) and even the design of hybrid bio-robots for biomedical applications (Xu *et al*., 2017).

The use of computers for flagellar tracking was pioneered by Hiramoto and Baba (1978), enabling the semi-automated capture of sperm flagellum [see Inaba and Shiba (2018) for an overview of the BohBoh system: BohBohSoft, Tokyo, Japan]. This software has been used for a number of studies of sperm hyperactivation (Ohmuro and Ishijima, 2006; Kaneko *et al*., 2007), for example, chemotaxis/chemokinesis (Shiba *et al*., 2006; Guerrero *et al*., 2010), and for the movement of other flagellated species such as *Leischmania* (Mukhopadhyay and Dey, 2016; Reddy *et al*., 2017). Our own group’s previous work has employed bespoke semi-automated algorithms (combined with significant manual input) to enable analysis of around 30–50 cells in studies of the effect of fluid rheology (Smith *et al*., 2009) and pharmacological stimulus (Ooi *et al*., 2014). Saggiorato *et al*. (2017) used a similar approach involving segmenting out cells and skeletonizing the resulting data to show that the flagellar waveform can be represented as the superposition of two bending waves, a fundamental frequency and a second harmonic. The authors suggest that modification of the relative phase between these two frequencies acts as a steering mechanism for sperm and other eukaryotic flagellates. Further recent methodological developments include the software of Walker and Wheeler (2018) and the SpermQ system (Hansen *et al*., 2018). We would, however, still characterize these techniques as requiring operator intervention (in particular manual segmentation) for the analysis of each set of imaging data, at least for the human sperm flagellum.

Outside of standard imaging and analysis procedures, there has been a growing interest in developing novel microfluidic devices for analysing the motility of sperm in controlled environments. The work of de Wagenaar *et al*. (2016) enables the trapping of single sperm cells and analysis of their beat frequency and amplitude using electrical impedance measurements. You *et al*. (2019) have recently developed their own sperm-trapping microfluidic device, utilizing Fourier analysis of image intensity to establish sperm beat frequency. These devices provide valuable tools for assessing sperm, and their ability to trap individual cells for analysis is of particular interest. While these devices are not necessarily aimed at clinical use (de Wagenaar *et al*., 2016), the technology does provide a potential complementary approach to the waveform analysis presented here for gaining detailed understanding about the motility of sperm.

There remains therefore a need to progress flagellar analysis methodology further for both basic research and clinical applications; semi-automated analysis can only reasonably enable tens of cells to be analysed in a reasonable time frame. This issue is particularly relevant to human sperm, which exhibit considerable heterogeneity (between cells, within cells over time, between ejaculates and between donors). It is therefore crucial to develop the tools to enable high-throughput flagellar analysis and thus take statistical measurements of flagellum movement over representative sample sizes.

This paper describes a free-to-use software package for high-throughput extraction and analysis of swimming sperm and their associated flagellar beat, Flagellar Analysis and Sperm Tracking (FAST). Using FAST, we have analysed 176 experimental microscopy videos and have tracked the head and flagellum of 205 progressive cells in diluted semen (DSM), 119 progressive cells in a high-viscosity medium (HVM) and 42 stuck cells in a low-viscosity medium. The average time to analyse each video was a few seconds per cell on an Apple Macbook Pro (Apple Inc., Cupertino, California). The package will be shown to provide positions in time of tracked sperm head and flagellum, and associated measurement of the flagellar arc-wavelength, flagellar beat frequency and power dissipation by the flagellum, in addition to providing the existing CASA measures. FAST is not designed to analyse raw semen; it is specifically for precise analysis of flagellar kinematics, as that is the promising area for computer use (Gallagher *et al*., 2018). Flagellar capture will always require that cells are at a dilution where their paths do not frequently cross. This manuscript reports detailed statistics on flagellar arc-wavelength and beat frequency and the metabolic requirements of motility, as well as the relationship of these measures to the standard CASA motility measures. We will discuss the calculated velocity profiles for some characteristic sperm calculated using a recently published open-source fluid mechanics code (‘NEAREST’; Gallagher and Smith, 2018; Smith, 2018; Gallagher *et al*., 2019). Additionally, we introduce a novel measure, track centroid speed (TCS), for differentiating progressive and non-progressive/immotile cells.

## Materials and Methods

Sperm flagellar movement exhibits major variations depending on the surrounding fluid environment and activation state. As such, the analysis has been performed on two data sets: (1) a new data set on free-swimming cells in two different viscosity media and (2) re-analysis of a previously published dataset showing the response of tethered cells to a hyperactivating pharmacological stimulus. Details of the two data sets follow.

### Analysis of free-swimming sperm in DSM and HVM

Semen samples were provided by three unscreened normozoospermic donors (recruited at Birmingham Women’s and Children’s NHS Foundation Trust after giving informed consent). Semen samples were obtained through masturbation following 2–3 days of abstinence. Cells were prepared according to two procedures in either DSM or HVM. To prepare the DSM, samples were counted according to WHO guidelines (WHO, 2010) and diluted to a concentration of 10 M/ml in supplemented Earle’s balanced salt solution (sEBSS) without phenol red, and supplemented with 2.5 mM Na pyruvate and 19 mM Na lactate (06–2010-03-1B; Biological Industries, Kibbutz Beit HaEmek, Israel), and 0.3% weight/volume charcoal delipidated bovine serum albumin (Sigma; A7906).

For HVM, cells were suspended in sEBSS without phenol red, and with the addition of pyruvate, lactate and bovine serum albumin, with the addition of 1% methylcellulose (M0512, Sigma-Aldrich, Poole, UK, specified so that an aqueous 2% solution gives a nominal viscosity of 4000 centipoise or 4 Pa s at ${20}^{{}^{\circ}}\mathrm{C}$).

For DSM cells were imaged in a 10 μm depth chamber, while HVM was loaded by capillary action into flat-sided borosilicate capillary tubes (VITROTUBES, 2540, Composite Metal Services, Ilkley, UK) with length 50 mm and inner dimensions 4 mm × 0.4 mm. One end of the tube was sealed with CRISTASEAL (Hawksley, Sussex, UK, #01503-00). Cells were selected for imaging by immersing one end of the capillary tube into a 1.5 ml Beem capsule (Agar Scientific, UK) containing a 200 μl aliquot of raw semen, within 30 minutes of sample production. Incubation was performed for 30 minutes at ${37}^{{}^{\circ}}\mathrm{C}$ in 6% CO2.

Both sets of cells were imaged using a Nikon (Eclipse 80i) microscope and negative phase contrast microscopy (objectives 10 × 0.25 Ph1 BM ∞/− WD 7.0 and 20 × 0.40 Ph1 BM ∞/0.17 WD 1.2), using a Basler Microscopy ace camera (acA 1300-200uc) at 169.2 frames per second with pixel size 4.8 μm × 4.8 μm, streaming data directly to a Dell XPS laptop using Pylon Viewer (v.5.0.11.10913, Basler AG, Ahrensburg, Germany). For DSM, the cells were imaged in 10 μm analysis chambers (10-01-04-B-CE; Leja Products B.V., Nieuw-Vennep, The Netherlands) at 10× magnification. Cells in HVM were imaged at 2 cm migration distance into the capillary tube, and in the surface accumulated layer 10–20 μm from the inner surface of the capillary tube at both 10× and 20× magnification.

### Re-analysis of adhered cells under pharmacological stimulus

For full details, see Ooi *et al*. (2014). Briefly, cells adhered to a surface coated with 0.1% poly-D-lysine in a bespoke chamber filled with supplemented saline solution were imaged at 20× magnification with negative phase contrast, before and after perfusion of 4-aminopyridine (4AP). Images were captured at 332 Hz using a Hamamatsu Photonics C9300CCD (Hamamatsu Photonics UK Ltd, Welwyn Garden City, UK.). Addition of 4AP has been shown be a strong inducer of hyperactivation in sperm by elevating intracellular calcium through release of calcium of stores in addition to channel activity (Alasmari *et al*., 2013). Hyperactivation of swimming sperm can be characterized by a high-amplitude bending in the flagellum and is important for improved migration and to aid in detachment from epithelial binding. Further, it has been shown that all of the CatSper sperm calcium ion channels that enable hyperactivated motility are required for normal fertility of mice (Quill *et al*., 2001; Quill *et al*., 2003; Qi et *al*., 2007). For these reasons, it is important to be able to correctly capture the significant change in sperm beat both before and after hyperactivation, making this data set a useful validator for FAST.

For each of experimental sets (1) and (2) the resulting imaging data was run through the FAST software package for tracking and analysis. In addition to the usual CASA kinematics obtained by tracking the position of the moving sperm head, FAST provides measurement of the flagellar arc-wavespeed (*c*), arc-wavelength (λ) and beat frequency (*f*).

### Computing resources and optics

FAST has been designed to work on Microsoft Windows, Apple Mac OS X and Linux-based operating systems, with minimal hardware requirements. All analysis for the present manuscript were performed on a machine with a quad-core Intel i7 processor and 16 GB of RAM although this is not the minimum required. FAST is designed to detect sperm contained in either audio video interleave (AVI) video files or tagged image format (TIF) image stacks, with any imaging modality where the entirety of the sperm cell is bright on a dark background.

### Flagellar kinematic parameter calculations

FAST characterizes the flagellar wave in terms of the tangent angle $\uptheta (\mathrm{s},\mathrm{t})$, a function of both the arclength along the flagellum *s* (measured in micrometers from the proximal end to the distal) and time *t*. We can then calculate the flagellar curvature as $ \kappa(\mathrm{s},\mathrm{t})=\mathrm{\partial \uptheta }/\mathrm{\partial s}$, from which the beat frequency (*f*) and arc-wavespeed $(c)$ can be derived, where we restrict the analysis region to be the section of the flagellum with $10\,\mu \mathrm{m}<\mathrm{s} < 30\,\mu \mathrm{m}$ as a region that ensures the wave has developed, is large enough to contain all the relevant information and is small enough to allow for fair comparisons between all cells.

#### Flagellar beat frequency

The flagellar beat frequency (*f*) is calculated by tracking the number of turning points of curvature for a choice of *s*, dividing by the time period *T* and then halving. This calculation is repeated for a number of points in *s* to reduce the effects of noise, and the median is taken as the final value for *f*.

#### Flagellar arc-wavespeed

To calculate the flagellar arc-wavespeed we begin, as for the beat frequency, by calculating the times corresponding to maxima in the curvature in time at *s* = 10 μm. Iterating forward in *s*, we follow the crest of each wave in time, achieving a set of $(\mathrm{s},\mathrm{t})$ pairs for each tracked wave. To fit the wavespeed to these points in a robust manner we formulate a linear mixed effects model (lme), consisting of a wave (straight line in $(\mathrm{s},\mathrm{t})$) with fixed effects on the slope and additional random effects on the slope and intercept to account for sperm metabolic noise inducing differences between waves. The output from solving this lme model not only gives a result for arc-wavespeed (*c*) and statistics about the goodness of fit, but also statistics about the random within-cell variation in wave speed.

#### Flagellar arc-wavelength

Having calculated the flagellar beat frequency (*f*) and arc-wavespeed (*c*), the flagellar arc-wavelength is then given by λ = *c*/*f*.

### Flagellar power dissipation calculations

Following Ooi *et al*. (2014), who applied the resistive force theory of Gray and Hancock (1955) [later modified by Lighthill (1976)], we write the hydrodynamic force exerted by the sperm flagellum per unit length as

$$ \mathrm{\textbf{f}} \left(\mathrm{s},\mathrm{t}\right)={\mathrm{K}}_{\mathrm{T}}\left(\mathrm{\textbf{u}}(\mathrm{s},\mathrm{t})\cdot \mathrm{\textbf{T}}(\mathrm{s},\mathrm{t})\right)\mathrm{\textbf{T}}\left(\mathrm{s},\mathrm{t}\right)+{\mathrm{K}}_{\mathrm{N}}\left(\mathrm{\textbf{u}}(\mathrm{s},\mathrm{t})\cdot \mathrm{\textbf{N}}(\mathrm{s},\mathrm{t})\right)\mathrm{\textbf{N}}
\left(\mathrm{s},\mathrm{t}\right), $$

where **u** is the velocity of the flagellum at arclength *s* and time *t*, **T** and **N** are the unit tangent and normal vectors to the flagellum and *K_T_* and *K_N_* are the tangential and normal resistance coefficients, where Lighthill’s ‘sub-optimal’ choice

$$ {\mathrm{K}}_{\mathrm{T}}=\frac{2\pi \mu}{\log 2q/a}\ \mathrm{and}\ {\mathrm{K}}_{\mathrm{N}}=\frac{4\pi \mu}{\log 2q/a+1/{2}}{\hbox{,}} $$

has been shown to be sufficiently accurate, with *μ* being fluid viscosity, *q* = 0.09λ and *a* being the typical radius of sperm flagellum. From this, the power dissipation due to the beating of a section of flagellum with ${s}_1<s<{s}_2$ can be calculated at a time *t* by

$$ \mathrm{P}=\int_{\mathrm{s}_1}^{\mathrm{s}_2}\mathrm{\textbf{f}}\left(\mathrm{s},\mathrm{t}\right)\cdot \mathrm{\textbf{u}}\left(\mathrm{s},\mathrm{t}\right)\mathrm{ds}. $$

### CASA measures

While the standard set of CASA measures have defined meanings, there are several differences in how the calculations are performed between many of the popular systems, not all of which are published. Here we will define each of the standard measures according to the WHO manual (WHO, 2010) and the methods by which FAST performs the calculations on head track locations ${\mathrm{\textbf{h}}}_{\mathrm{i}}=({\mathrm{x}}_{\mathrm{i}},{\mathrm{y}}_{\mathrm{i}})$ at times ${\mathrm{t}}_{\mathrm{i}}$, at *n* + 1 points, $\mathrm{i}=1,\dots, \mathrm{n} + 1$.

VCL: time averaged curvilinear velocity in μm/s,

$$ \mathrm{VCL}=\frac{1}{n} \sum_{\mathrm{i}=1}^{\mathrm{n}}\frac{{\|\mathrm{\textbf{h}}}_{\mathrm{i}+1}\, - \, {\mathrm{\textbf{h}}}_{\mathrm{i}}\|}{{\mathrm{t}}_{\mathrm{i}+1}\,
- \, {\mathrm{t}}_{\mathrm{i}}}. $$

VSL: straight-line velocity in μm/s,

$$ \mathrm{VSL}=\frac{{\|\mathrm{\textbf{h}}}_{\mathrm{n}+1}\, -\, {\mathrm{\textbf{h}}}_{\mathrm{1}}\|}{{\mathrm{t}}_{\mathrm{n}+1}\,
- \, {\mathrm{t}}_1}. $$

VAP: average path velocity in μm/s. In FAST, we construct a robust average path by calculating a wavelet decomposition at level 7 of the path $\mathrm{\textbf{h}}$ using a discrete Meyer wavelet. We then discard the detail levels 1–4, and rebuild the path using wavelet reconstruction, giving the points ${\mathrm{\textbf{A}}}$.

ALH_max_: maximum amplitude of lateral head displacement in μm. Maximum displacement of the head track about the average path,

$$ {\mathrm{A}\mathrm{LH}}_{\mathrm{max}}=\underset{\mathrm{i}=1,\dots, \mathrm{n}+1}{\max}\left\Vert {\mathrm{\textbf{h}}}_{\mathrm{i}}\,-\,{\mathrm{\textbf{A}}}_{\mathrm{i}}\right\Vert . $$

ALH_avg_: average amplitude of lateral head displacement in μm,

$$ {\mathrm{A}\mathrm{LH}}_{\mathrm{avg}}=\frac{1}{\mathrm{n}+1}\sum_{\mathrm{i}=1}^{\mathrm{n}+1}\left\Vert {\mathrm{\textbf{h}}}_{\mathrm{i}}\,-\,{\mathrm{\textbf{A}}}_{\mathrm{i}}\right\Vert . $$

LIN: linearity of the curvilinear path. LIN = VSL/VCL.

WOB: wobble, a measure of oscillation of the curvilinear path about the average path. WOB = VAP/VCL.

STR: straightness of the average path. STR = VSL/VAP.

BCF: beat cross frequency in Hz, the rate at which the curvilinear path crosses the average path. To calculate BCF, we rotate each segment of $\mathrm{\textbf{h}}$ and $\mathrm{\textbf{A}}$ to ${\bar{\mathrm{\textbf{h}}}}$ and $ {\bar{\mathrm{\textbf{A}}}}$ so that the rotated average path segment ${\bar{\mathrm{\textbf{A}}}}$ lies along the horizontal axis, sum the number of changes in sign in ${\bar{\mathrm{\textbf{h}}}}$ and divide by the time period.

MAD: mean absolute angular displacement in degrees,

$$ \mathrm{MAD}=\frac{1}{\mathrm{n}}\sum_{\mathrm{i}=1}^{\mathrm{n}}\left| {\tan}^{-1}\left(\frac{{\mathrm{y}}_{\mathrm{i}+1}\, -\, {\mathrm{y}}_{\mathrm{i}}}{{\mathrm{x}}_{\mathrm{i}+1}\,-
\,{\mathrm{x}}_{\mathrm{i}}}\right)\right| . $$

TCS: track centroid speed. We have also proposed the introduction of a new CASA measure for differentiating progressive cells, the TCS, calculated as

$$ \mathrm{TCS}=\frac{1}{\left({\mathrm{t}}_{\mathrm{n}+1}-{\mathrm{t}}_1\right)\left(\mathrm{n}+1\right)}\left\Vert \sum_{\mathrm{i}=1}^{\mathrm{n}+1}\left({\mathrm{\textbf{h}}}_{\mathrm{i}}-{\mathrm{\textbf{h}}}_1\right)\right\Vert . $$

### Statistical analyses

Statistical analyses surrounding the relationship between directly measured flagellar beat frequency and BCF consisted of line fitting in MATLAB, with the *R*^2^ values reported being the ratio of the sum of squares of the regression to the total sum of squares.

### Parameters for FAST

FAST parameters used in the analysis of the data in this manuscript can be found in the spreadsheets of data in Supplementary Tables S1–S3.

### Ethical approval

All donors were recruited in accordance with the Human and Embryology Authority Code of Practice (version 7) and gave informed consent (South Birmingham LREC 2003/239 and East Midlands REC 13/EM/0272).

## Results

**Figure 1:**
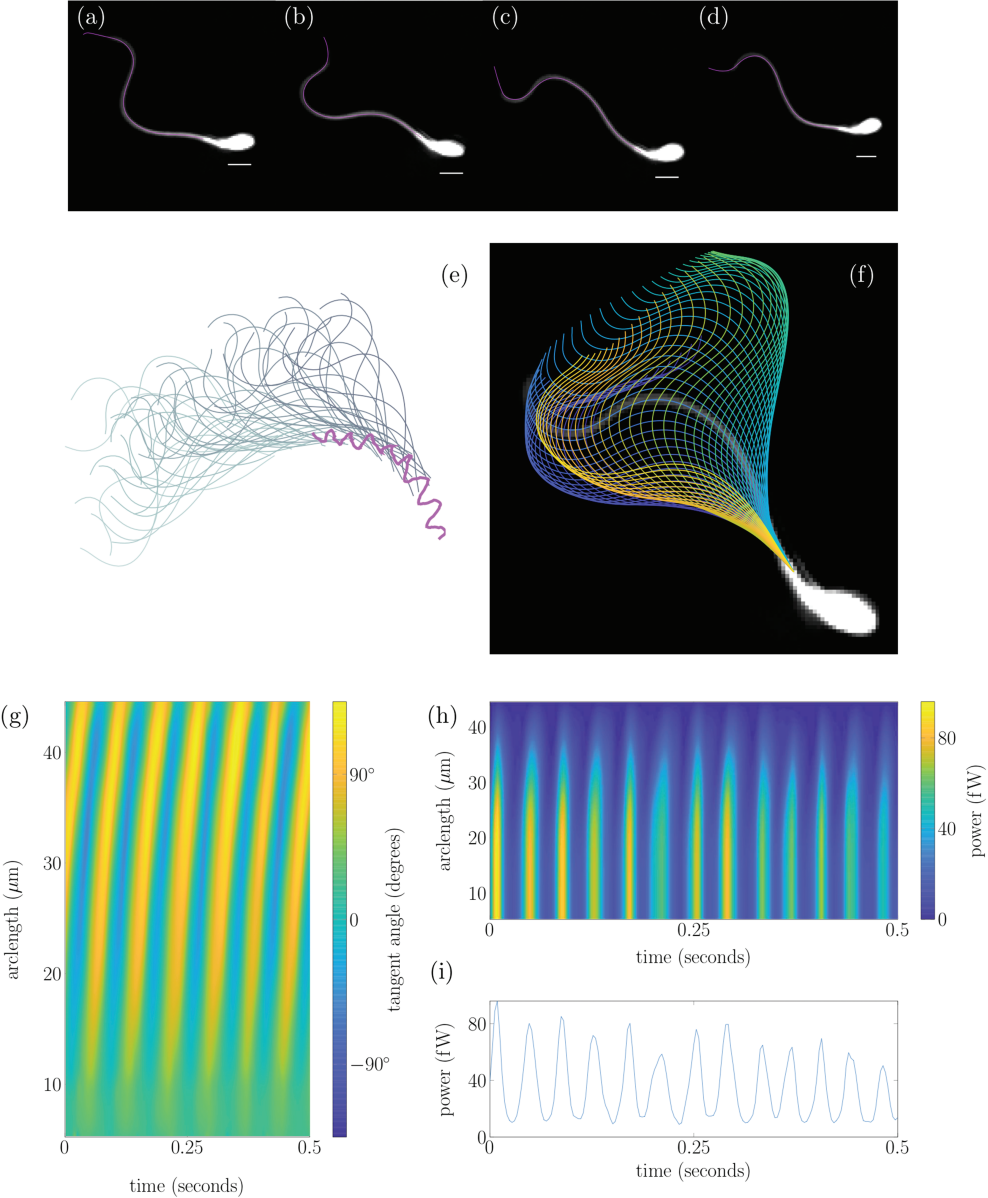
**Tracking of a human sperm from experimental data set (1) in the DSM medium.** Panels (**a**)–(**d**) show an overlay of the tracked flagellum over experimental frames at four points in a beat cycle, with a 5 μm white scale bar. Panel (**e**) shows the sperm head track in magenta with associated flagellum plotted 0.014 s apart. Panel (**f**) plots the flagellar beat over a single experimental frame with the colour of each flagellum representing time from dark blue to yellow. Panel (**g**) plots the tangent angle along the flagellum in the cell frame for 0.5 s. Panel (**h**) shows the power exerted by the flagellum on the fluid distal to the point in arclength chosen, with the power exerted by the full flagellum shown in panel (**i**).

**Figure 2:**
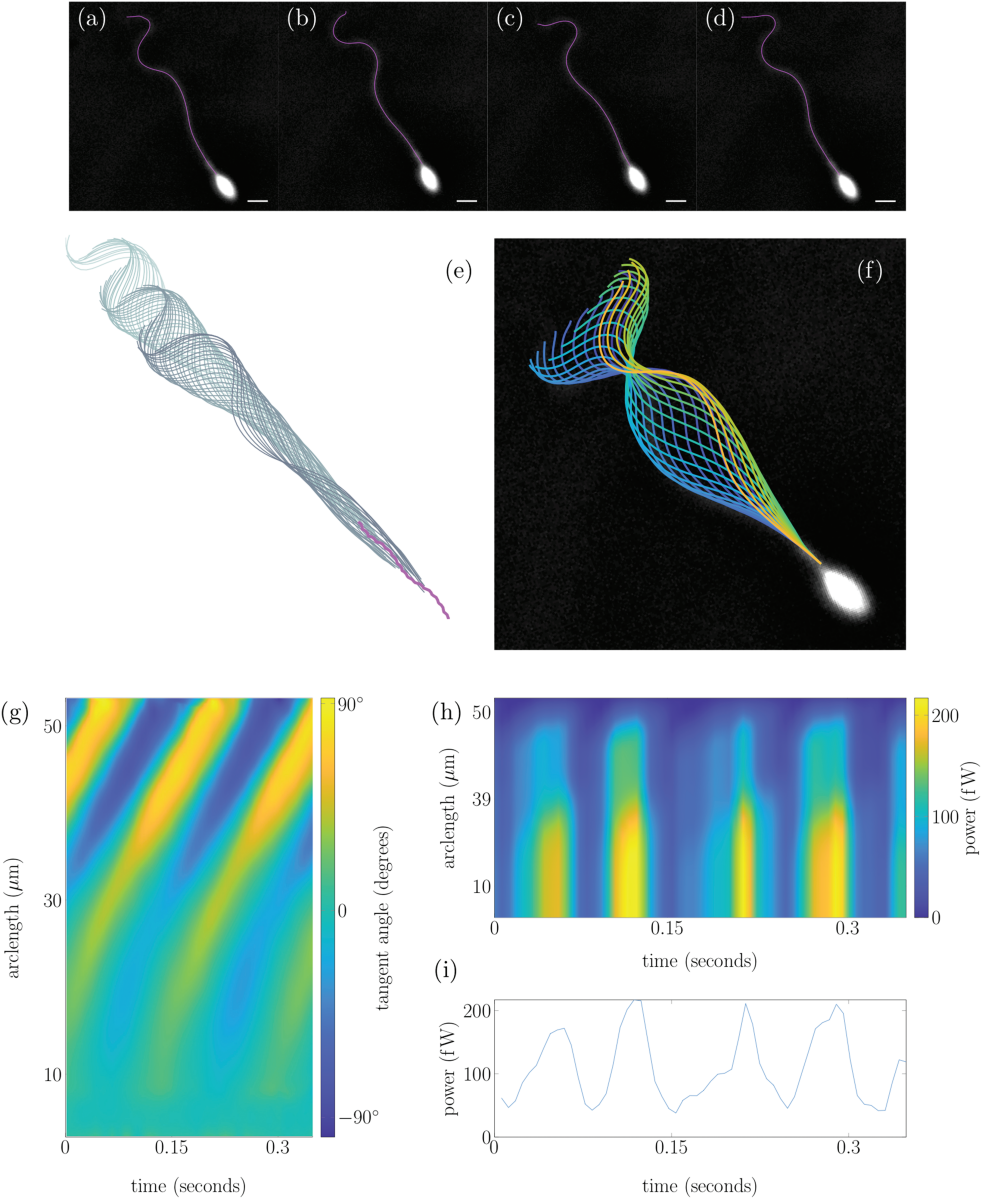
**Tracking of a human sperm from experimental data set (1) in the HVM.** Panels (**a**)–(**d**) show an overlay of the tracked flagellum over experimental frames at four points in a beat cycle, with a 5 μm white scale bar. Panel (**e**) shows the sperm head track in magenta with associated flagellum plotted 0.007 s apart. Panel (**f**) plots the flagellar beat over a single experimental frame with the colour of each flagellum representing time from dark blue to yellow. Panel (**g**) plots the tangent angle along the flagellum in the cell frame for 0.35 s. Panel (**h**) shows the power exerted by the flagellum on the fluid distal to the point in arclength chosen, with the power exerted by the full flagellum shown in panel (**i**).

**Figure 3:**
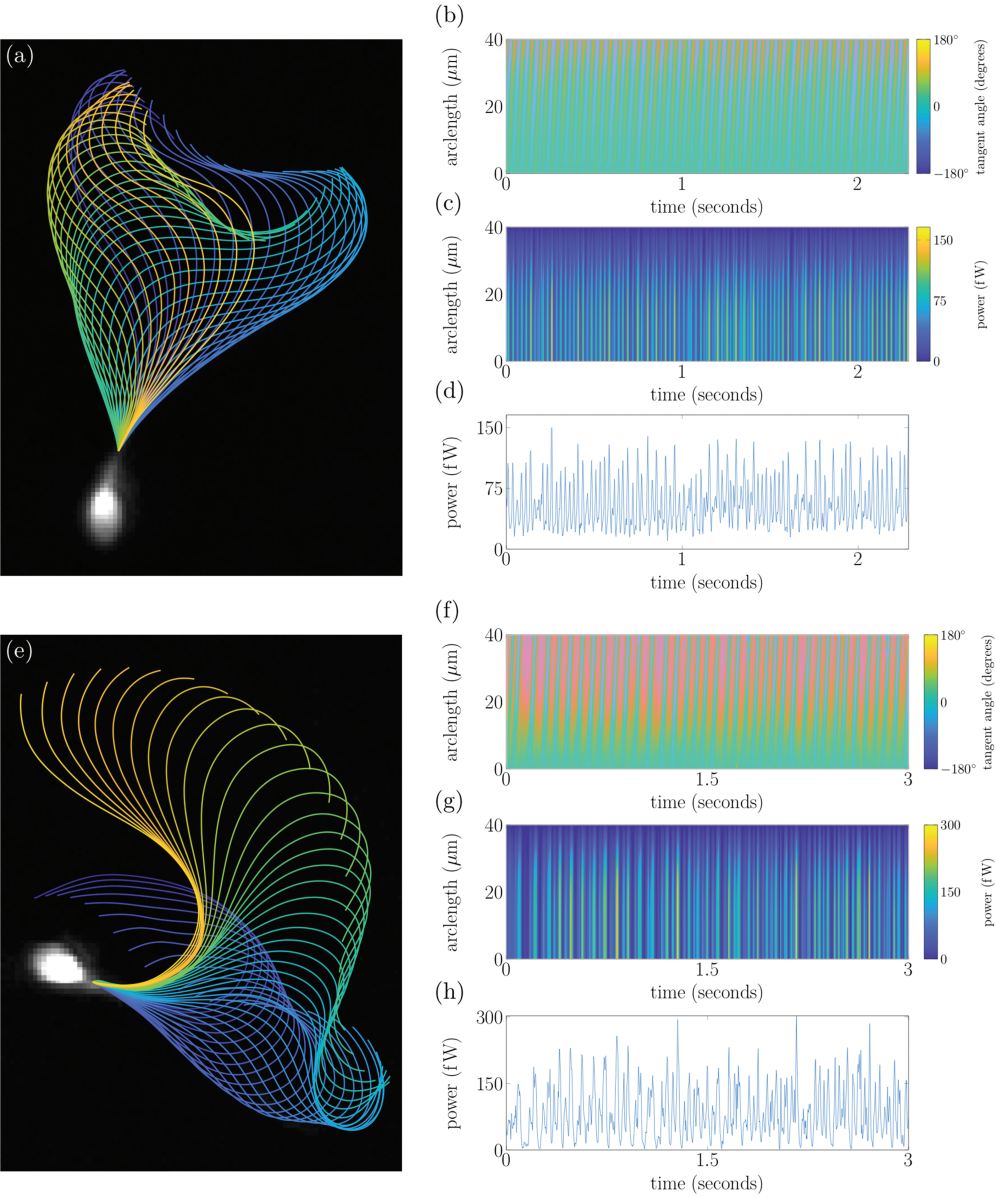
**Tracking of stuck sperm from experimental data set (2) enabling long-time analysis of cells.** Panels (**a**)–(**d**) show the flagellar beat, tangent angle, power exerted by the flagellum distal to a point in arclength and total power exerted by the flagellum, respectively. Panels (**e**)–(**h**) show the same plots for a hyperactivated cell after stimulation with 4AP.

The results of the analysis of experimental sets (1) and (2) (for some characteristic sperm) are shown for (i) free-swimming cells in Figs 1 and 2 (DSM and HVM, respectively) and for (ii) adhered cells before and after addition of 4AP in Fig. 3. The videos used for the images in this manuscript and analysed data points are Supplementary Videos S1–S4.

**Figure 4:**
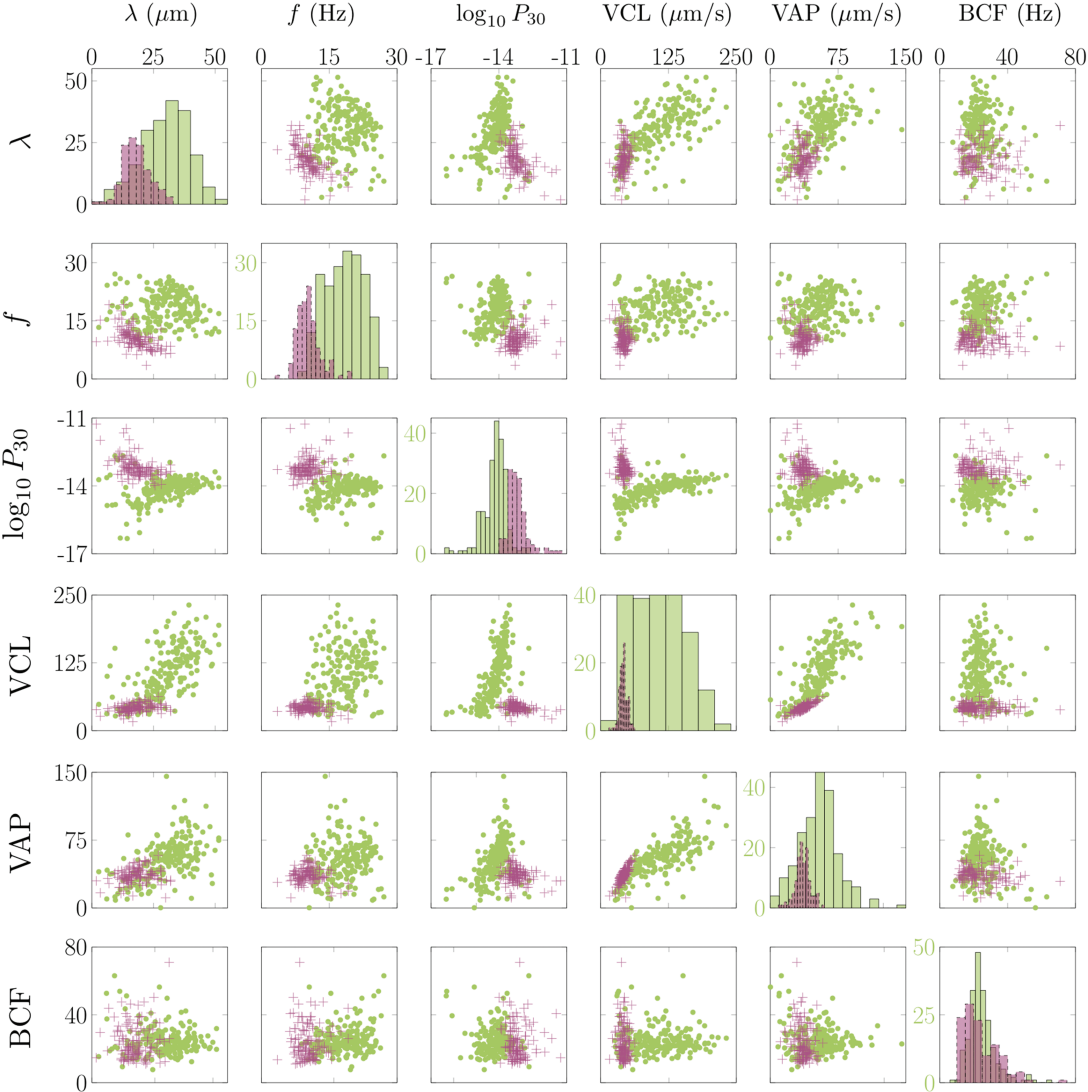
**Scatter plot matrix.** Scatter plot matrix showing relationships between arc-wavelength λ, flagellar beat frequency f, power generated by the first 30 μm of flagellum P_30_ (measured in watts and plotted on a log scale), the curvilinear velocity of the head (VCL), the average path velocity of the head (VAP) and the beat cross frequency of the head (BCF). Axes persist from left to right and top to bottom except on the leading diagonal where frequencies are shown in green. In each plot, sperm swimming through HVM are shown as magenta crosses and sperm swimming through DSM are shown as green dots.

For experimental set (1) we plot λ, *f*, power dissipation and three of the established CASA measures [VCL, VAP and BCF] for each cell tracked in a scatter plot matrix (Fig. 4). The distinction between motilities in DSM and HVM is immediately apparent, forming two distinct sub-populations in the data. Notably, sperm in DSM have a median arc-wavelength of 31 μm with interquartile range (IQR) of 13 μm, decreasing down to a median arc-wavelength of 17 μm with IQR of 7 μm in HVM. Similarly, cells in DSM have a median beat frequency of 19 Hz with IQR of 6.5 Hz, decreasing to a median beat frequency of 10 Hz with an IQR of 2.9 Hz in HVM. Note that, in this manuscript, we are using the Lighthill (1975) notion of arc-wavespeed and arc-wavelength rather than the conventional wavespeed and wavelength. This is due to the variation in the axis of wave propagation for sperm flagellum, making measurements in terms of the arclength along the flagellum a more natural choice.

### Metabolic requirements of motility from imaging data

Knowledge of the flagellar waveform allows, via fluid dynamic modelling, for calculation of the power dissipation due to the beating of the flagellum. There are many methods to do this, each providing a balance between accuracy and ease-of-implementation (Gaffney *et al*., 2011; Gallagher and Smith, 2018; Smith, 2018). After analysis with FAST, the captured flagellar movement can be used as input to a fluid mechanics calculation, which can then be used to assess the hydrodynamic power dissipation along the flagellum as demonstrated by Ooi *et al*. (2014). In the present work, we treat the surrounding fluid as Newtonian allowing the application of resistive force theory (Gray and Hancock, 1955; Lighthill, 1976; Gueron and Liron, 1992) (details in the methods section), which is acceptably accurate for the analysis of sperm motility (Johnson and Brokaw, 1979; Friedrich *et al*., 2010). Recently, modifications of resistive force theory have been suggested for non-Newtonian fluids (Riley and Lauga, 2017), which would enable the use of tracking data in modelling behaviours in more biologically complex fluids.

In Figs 1i, 2i, 3d and 3h, we plot the total power dissipation in watts integrated along the flagellum against time for a typical cell swimming in our low- and high-viscosity media, stuck and stuck and stimulated with 4AP, respectively. To understand how the power dissipation varies along the flagellum in Figs 1h, 2h, 3c and 3g, we plot the time-dependent power dissipation distal to a point in arclength along the flagellum. For fair comparison between cells when a varying length of flagellum is visible in experiments (often due to cell rolling or significant out-of-plane beating), we calculate the power dissipation by the first 30 μm of flagellum (P_30_, shown with comparison to other measures in Fig. 4). The cells tracked in DSM reveal a median P_30_ of ~8.4 femtowatt (fW) with IQR of 9.4 fW (note: 1 fW is equal to 10^−15^ watts). In contrast the cells swimming in HVM have an increased median *P*_30_ of 59 fW with IQR of 61 fW. For validation of the software package, we have reanalysed the Ooi *et al*. (2014) data, which reported a median 7% decrease in cell power after the addition of 4AP; the reanalysis of a subset of these cells revealed a commensurate 19% decrease in the value of P_30_, where it should be noted exact comparisons of power dissipation over the full length of flagellum cannot be made due to the increased length of captured flagellum by FAST.

### Relationship between traditional CASA measures and sperm flagellar kinematics

In Fig. 4, we show comparisons between the flagellar arc-wavelength (λ) flagellar beat frequency (*f*), and the power dissipation through the first 30 μm of flagellum P_30_, as well as three of the commonly used existing CASA measures, namely VCL, VAP and BCF. The change in media from DSM to HVM defines two clear clusters in the data.

#### BCF does not measure flagellar beat frequency

BCF (the frequency with which the curvilinear path of the sperm head crosses the average path) was introduced as a proxy for flagellar beat frequency as it was impossible to make the correct measurement (Mortimer, 1997). We have been able to assess this quantitatively, with the results plotted in Fig. 4. Attempting to fit to the data, we obtain *R*^2^ values of 0.042 and 0.00054 for cells in DSM and HVM, respectively, showing that BCF does not measure the flagellar beat frequency.

#### Flagellar kinematics provide much stronger separation of different motility modes than CASA

Assessment of the data, as seen in the histograms in Fig. 4, reveals that the flagellar kinematic measures show greater separation between sperm in DSM versus HVM than do standard CASA parameters. This not only emphasizes the dramatic differences in sperm motility in different media, but also the importance of being able to accurately characterize them.

### New insights into sperm dynamics with modelling

**Figure 5:**
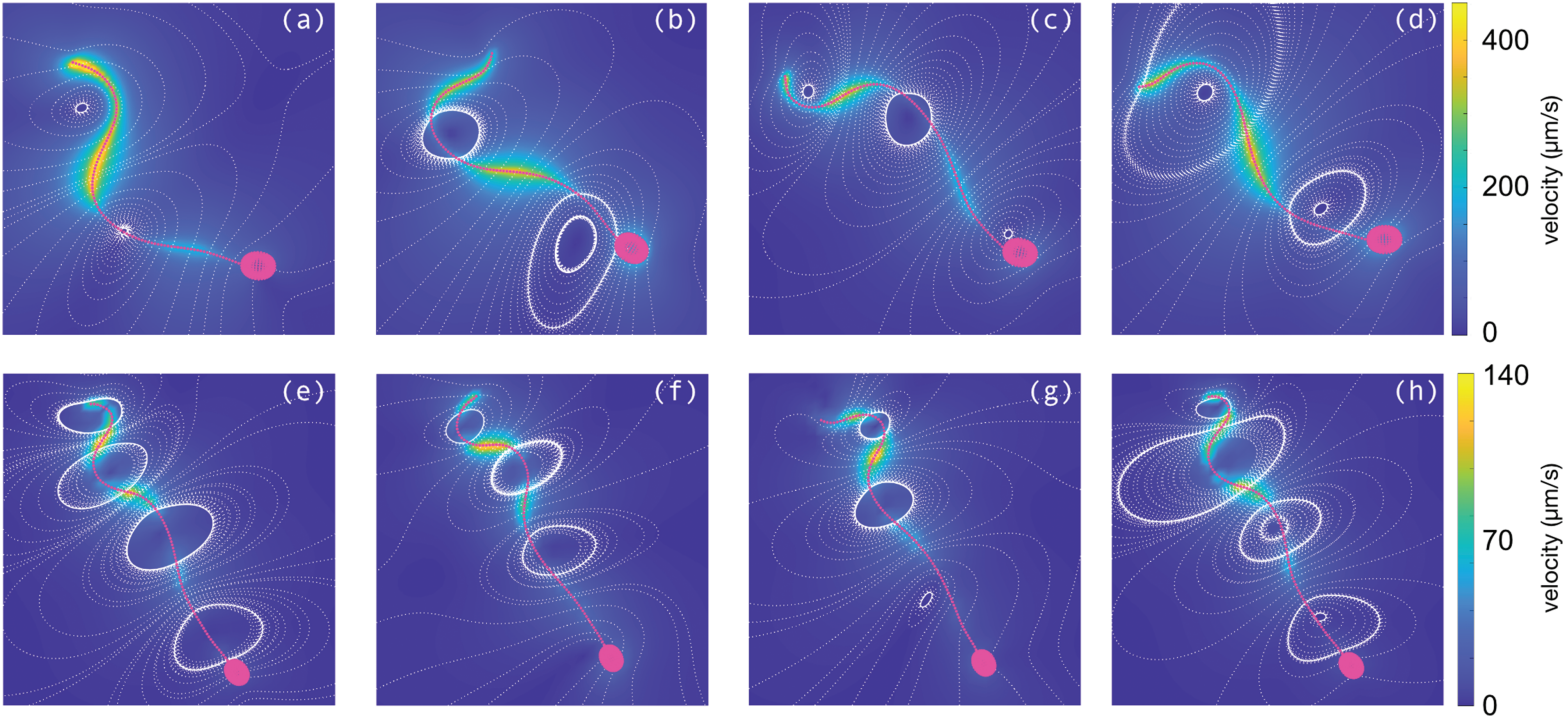
**Simulated velocity fields with NEAREST for the tracked sperm in Figs 1 and 2**. The FAST tracked flagellum for each sperm has been paired with an idealized head and simulated in a three-dimensional environment. Panels (**a**)–(**d**) show the flow fields in DSM at times corresponding to Fig. 1a–d, while (**e**)–(**h**) are in HVM, corresponding to Fig. 2a–d. In each figure the colour depicts the fluid velocity magnitude, with the sperm cell overlain in magenta and streamlines shown as white dots.

The wealth of data provided by accurately tracking the sperm flagellar beat provides an exciting opportunity for use with mathematical models to gain new insights into the fluid dynamic properties induced by sperm swimming. Previously, to visualize the fluid velocity field surrounding a swimming cell required the use of experimental techniques such as micro-particle image velocimetry (Hamad, 2017), which is time consuming and prone to error. Instead we are able to pass an extracted flagellar beat, together with a model of a sperm head, to the NEAREST code package (Gallagher and Smith, 2018; Smith, 2018; Gallagher *et al*., 2019). By solving the resulting fluid dynamics problem for a swimming sperm, we are able to calculate the fluid velocity field surrounding the cell, as plotted in Fig. 5. Here we see that there are significant differences in the velocity fields between the typical DSM and HVM cells, with the swimming of the cell in DSM inducing a much greater change in the fluid velocity due to the beating of the flagellum.

### A new way to categorize progressive sperm

**Figure 6:**
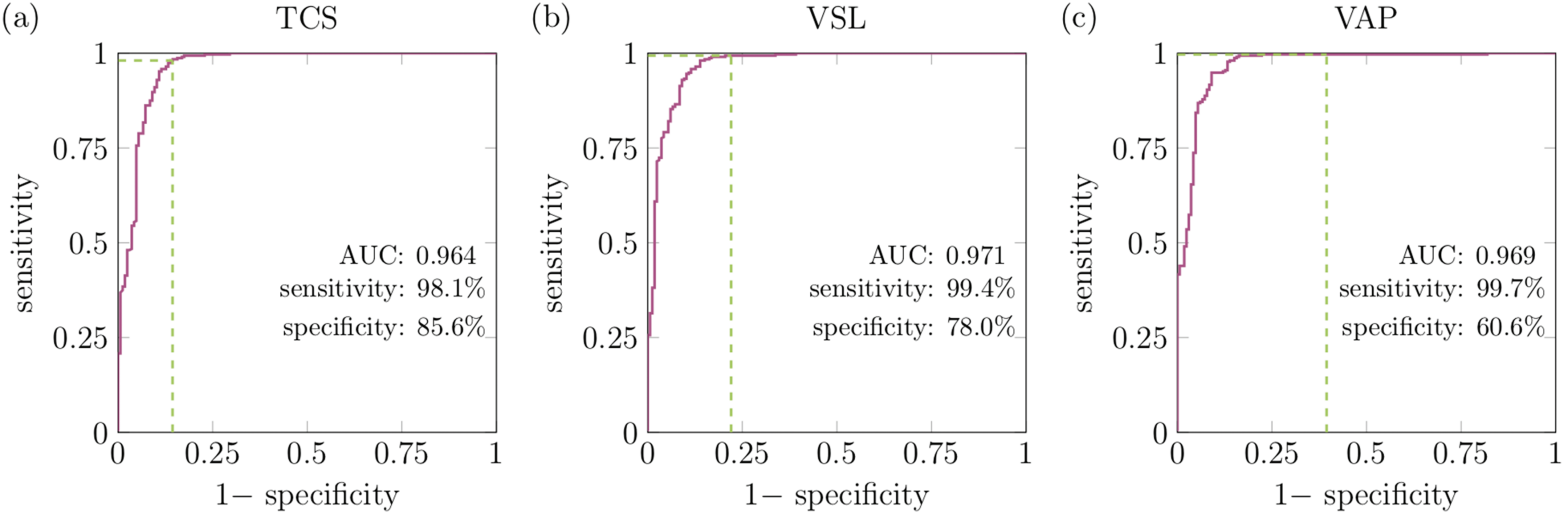
**ROC curves for characterizing sperm as progressive compared to the gold standard manual classification by a trained analyst.** In panel (**a**) the ROC curve using TCS is plotted, with the more standard use of VSL and VAP shown for comparison in panels (**b**) and (**c**). The green lines highlight the sensitivity and specificity when using the 5 μm/s WHO categorization (WHO, 1999).

The WHO-IV (WHO, 1999) threshold for classifying a cell as progressive is a velocity greater than 5 μm/s; however, there is no consensus on which of the established CASA velocity measures should be used for this classification. VSL (straight line velocity from first point tracked to last) may be misleading when sperm motility is circular as it has a significant dependence on the length of time the cell is tracked for, while the use of VCL and VAP may result in misclassification of cells that are twitching or being buffeted in tight circles as being progressive. We propose a new measure, the TCS (defined in the methods section), for this purpose. In Fig. 6, we plot the receiver operating characteristic (ROC) curves for characterizing progressive sperm using TCS, as well as the standard CASA measures of VSL and VAP, against the gold standard of manual classification by a trained analyst. For each curve, we see a significant area under the curve of between 0.964 and 0.971, with similar sensitivities of 98.1–99.7% (with TCS taking the lower of each of these measures). Where TCS truly outperforms the use of VSL and VAP is in the specificity of 85.6% compared to 78% and 60.6% for VSL and VAP, respectively.

## Discussion

Meaningful clinical information in a heterogeneous population requires analysis of large cell numbers. Although the sperm flagellar waveform contains a wealth of potential diagnostic power, until now this could not be harnessed as the necessary tools were unavailable. This heterogeneity of human sperm kinematic motion means that excellent studies, which have been performed on small numbers of cells, have only been able to hint at underlying mechanisms and relationships, such as the link between sperm velocity and flagellar movement. Furthermore, there is a clear desire for new tools to assess large quantities of cells for human fertility research as well as male reproduction more generally. The FAST software package provides this ability, enabling assessment of large numbers of individual cells and the statistical power necessary to understand sperm motility on the population scale.

Rapid sperm motility is crucial for natural fertilization (Holt *et al*., 1985; Hirano *et al*., 2001), and the diagnostic accuracy of this should improve with the availability of flagellar beat analysis. Developing a correct understanding of sperm motility will provide a potential diagnostic tool for male health more generally, and a substantial base for therapeutic developments. To address this we have developed and released FAST, the free-to-use software package for high-throughput flagellar beat analysis and cell tracking. We have tracked and analysed 366 sperm. By describing the flagellum as a tangent angle formulation, we are able to provide detailed reporting of flagellar kinematic measures, such as beat frequency and arc-wavelength for individual and populations of cells. In addition, the software provides improved accuracy for the existing CASA measures of motility. In particular, these data highlight that the existing BCF parameter is an incorrect extrapolation of flagellar beat frequency. In the present manuscript, we have focused on 2D imaging exploiting the largely planar nature of the beat of the human sperm; however, advances in imaging are increasingly making it possible to take three-dimensional scans of swimming microorganisms (Pimentel *et al*., 2012; Su *et al*., 2012). It would be of interest, particularly for species other than human, to extend the capabilities of the software for non-planar flagellar motion.

Combining tracked flagella with mathematical modelling has the potential to reveal new mechanistic insight; for example, it is now possible to estimate the metabolic requirements of motility, a further quantity that is not accessible when only the head of a cell is tracked. Particle-based methods to image flow on microscopic scales are technically challenging and limited in resolution due to Brownian effects. In contrast, the combination of negative phase contrast microscopy, FAST and the NEAREST fluid dynamics package (Gallagher and Smith, 2018) provide a much more convenient, rapid and highly resolved (if indirect) method to estimate fluid dynamic effects, such as that of a swimming cell on its surrounding environment. We have designed FAST to be agnostic to fluid mechanics, enabling the coupling of tracked analysis with more advanced computational methods (for example, incorporating non-Newtonian effects), as they become available.

The novel ability to quantify accurate flagellar beat detail in large populations of motile cells enables an abundant array of diagnostic, toxicological and therapeutic possibilities. In particular, we hope that it opens the way for new approaches to assessing and treating male subfertility.

## Supplementary Material

Supplementary_data_dez056
