# Supplementary material for: Rapid sperm capture: high-throughput flagellar waveform analysis
Source: Hum Reprod. 2019 Jun 7;34(7):1173–85. doi: 10.1093/humrep/dez056 (PMC6613345; doi:10.1093/humrep/dez056)
Supplement: Supplementary_data_dez056 [file supplementary_data_dez056.zip › Supplementary_data_dez056.pdf]

Human Reproduction, pp. 1–1, 2019
doi:10.1093/humrep/dez056

human reproduction

SUPPLEMENTARY DATA

Supplementary Table SI Output statistics from FAST for the swimming cells from experimental set (1), a selection of which is plotted in Figure 5.

Supplementary Table SII Output statistics from FAST for the adhered cells from experimental set (2).

Supplementary Table SIII Manually assessed points for ROC curve analysis presented in Figure 6.

Supplementary Video 1 Video of sperm swimming in diluted serum medium (DSM), used for the images in Figure 1.

Supplementary Video 2 Video of sperm swimming in high viscosity medium (HVM), used for the images in Figure 2.

Supplementary Video 3 Video of adhered sperm with no external stimulii, used for the images in Figure 3, panels (a)-(d).

Supplementary Video 4 Video of adhered sperm, stimulated with 4AP, used for the images in Figure 3, panels (e)-(h).
